# A Plot Twist: When RNA Yields Unexpected Findings in Paired DNA-RNA Germline Genetic Testing

**DOI:** 10.3390/genes16111382

**Published:** 2025-11-13

**Authors:** Heather Zimmermann, Terra Brannan, Colin Young, Jesus Ramirez Castano, Carolyn Horton, Alexandra Richardson, Bhuvan Molparia, Marcy E. Richardson

**Affiliations:** Ambry Genetics, 1 Enterprise Drive, Aliso Viejo, CA 92656, USA

**Keywords:** RNA, splicing, genetic testing, spliceosome, aberrant splicing, alternative splicing, variant interpretation

## Abstract

Background: Germline genetic variants impacting splicing are a frequent cause of disease. The clinical interpretation of such variants is challenging for many reasons including the immense complexity of splicing mechanisms. While recent advances in splicing algorithms have improved the accuracy of splice prediction, predicting the nature and abundance of aberrant splicing remains challenging. As RNA testing becomes more mainstream in the clinical diagnostic setting, the complexities of interpretation are coming to light. Methods: Data from patients undergoing concurrent DNA and RNA testing were retrospectively reviewed for unusual splicing impacts to underscore some of these complexities and serve as exemplars in how to avoid pitfalls in the interpretation of sequence variants. Results: Seven rare variants with unusual splicing impacts are presented: a variant at a consensus donor nucleotide position lacking a splice impact (*NF1* c.888+2T>C); a mid-exonic missense variant creating a novel donor site and a cryptic acceptor site resulting in pseudo-intronization (*BRIP1* c.727A<G p.Ile243Val); one variant creating a spliceosome switch from U12 to U2 (*LZTR1* c.2232G>A p.Ala744Ala); two variants that would be expected to result in nonsense-mediated-mRNA-decay triggering splicing impacts that obviated nonsense-mediated-decay (*APC* c.1042C>T p.Arg348Ter and *BRCA2* c.6762del; c.6816_6841+1534del); and two variants causing splicing impacts through pyrimidine tract optimization (*NF1* c.5750-184_5750-178dup and *ATM* c.3480G>T p.Val1160Val). Conclusions: Paired DNA and RNA testing revealed unexpected splice events altering variant interpretation, expanding our knowledge of clinically important splicing mechanisms and highlighting the benefit of RNA testing.

## 1. Background

Germline genetic variants that result in aberrant mRNA splicing are estimated to constitute up to 10% of clinically relevant alterations [1,2]. The clinical interpretation and classification of spliceogenic variants can be challenging for many reasons, including the volume of potentially spliceogenic variants, a lack of comprehensive clinical and functional data, and the challenges around predicting the nature and abundance of aberrant splicing given the immense complexity of normal splicing mechanisms [3]–often resulting in variants of uncertain significance (VUS).

Nascent RNA molecules undergo a maturation process that involves 5′ end capping, removal of introns, and 3′ polyadenylation making it ready for efficient translation by the ribosomes. The process of removing introns from pre-mRNA is called splicing and it involves cleavage and joining at a 5′ donor site and a 3′ acceptor site effectively removing the intron and joining exons. RNA splicing is catalyzed by the spliceosome which is a dynamic macromolecular machine that has been studied for decades. However, there remains much to be understood about how it so precisely recognizes and uses a wide variety of splice signals in a context-specific manner. Intron recognition, which is fundamental to the splicing process, occurs through conserved motifs at exon-intron junctions that are detected by components of the spliceosome. Such recognition motifs include (1) a splice donor site at the 5′ end of the intron; (2) a splice acceptor site at the 3′ end of the intron encompassing the consensus sequence region, a branch point (typically an adenine residue approximately 20 nucleotides upstream of the exon), and a polypyrimidine tract of variable length and nucleotide composition; and (3) relatively poorly understood exonic or intronic splice enhancer and inhibitor motifs [4].

Sequence changes that disrupt the conserved splicing motifs at native splice sites or that activate cryptic motifs can lead to aberrant splicing. The identification of genetic variants that cause aberrant splicing is informative for identifying familial risk through cascade testing as well as for identifying targets for antisense oligonucleotide therapies [5]. Such aberrant splicing causes the exclusion of exonic material or inclusion of intronic sequences into the coding sequence, resulting in nonsense-mediated decay (NMD) or abnormal protein [2]. NMD is the mechanism by which an RNA transcript containing a premature termination codon (PTC) is degraded, leading to haploinsufficiency and pathogenicity in genes where loss-of-function is a mechanism of disease [6].

The majority of known, clinically relevant spliceogenic variants involve changes to the first two (+1 and +2) or last two (−1 and −2) nucleotide positions of an intron, often called the canonical splice sites [1,2]. Canonical splice site variants are presumed to evoke a complete or near-complete splice impact and can be given a very strong line of a priori evidence towards pathogenicity [7,8,9,10]. Coded as Pathogenic Very_Strong 1 code (PVS1) under the American College of Medical Genetics/American College of Medical Genetics (ACMG/AMP) guidelines, this very strong line of evidence can be applied for a canonical variant in loss-of-function genes when the splice impact is predicted to result in NMD.

Alterations outside of the canonical positions, including mid-exonic and deep-intronic variants, may also impact splicing in a clinically relevant way. However, generalizing their impact is more difficult given the variability that is tolerated at these positions and our limited understanding of what is required for the activation of novel splice sites. Variants outside of the canonical positions may cause an incomplete splice impact in which both aberrant and wild-type transcripts derive from the altered allele. Variant interpretation for such variants relies heavily on other lines of variant interpretation evidence including clinical data, functional data, and in silico data.

The recent incorporation of machine learning methods into in silico splice prediction algorithms such as SpliceAI [11] has significantly improved the prediction of splice effects. While these tools are good at predicting whether a variant strengthens or weakens a given splice site, interpreting the nature and completeness of the associated splice impact adds additional layers of complexity and warrants further RNA analysis and interpretation.

In this work, we present seven instances where RNA studies revealed unexpected splicing impacts or unusual splicing mechanisms that would have been difficult to predict a priori. Although final variant interpretation relies on many evidence types and is outside the scope of this work which focuses solely on RNA findings, these data highlight the importance of including RNA in a clinical diagnostic test to form a comprehensive picture of the nature of every variant.

## 2. Methods

### 2.1. Testing Cohort

Results from over 200,000 individuals submitting samples for paired DNA-RNA genetic testing at a single diagnostic laboratory (Ambry Genetics, Aliso Viejo, CA, USA) between 2020 and 2023 were retrospectively analyzed. Demographics and clinical data were provided by the ordering provider as part of standard diagnostic testing.

### 2.2. CaptureSeq RNA Testing and RT-PCRseq

Full gene DNA sequencing, deletion and duplication analysis, and RNA analysis were performed as described previously [12,13]. Briefly, CaptureSeq analysis was performed on cDNA from whole blood using standard clinical cancer panel libraries. Results were compared to thousands of clinical control samples that do not carry the same variants. Where warranted, reverse transcriptase polymerase chain reaction sequencing (RT-PCRseq) was run on the same samples with custom primers (sequences available upon request) and compared to same-run control sample(s) that do not contain a reportable variant in the same gene. Percent Splicing Index (PSI) is calculated from reads as previously described. Where possible, allele-specific expression of informative variants is considered if another variant is in-range and contained within the transcript.

## 3. Results

### 3.1. Canonical Variant with No Abnormal Splicing

Recent studies have shown that an estimated 15–18% of T>C transitions at the +2 position generate a variable amount of wild-type (WT) transcripts [14,15]. This is inconsistent with the assumption of a complete splice defect that is typically applied to canonical splice site substitutions and could lead to the incorrect application of the ACMG/AMP Variant Interpretation Guidelines for PVS1 [7,16]. Here, we describe a +2T>C change that generates predominantly wild-type transcripts and does not appear to cause a disease phenotype.

**Figure 1:**
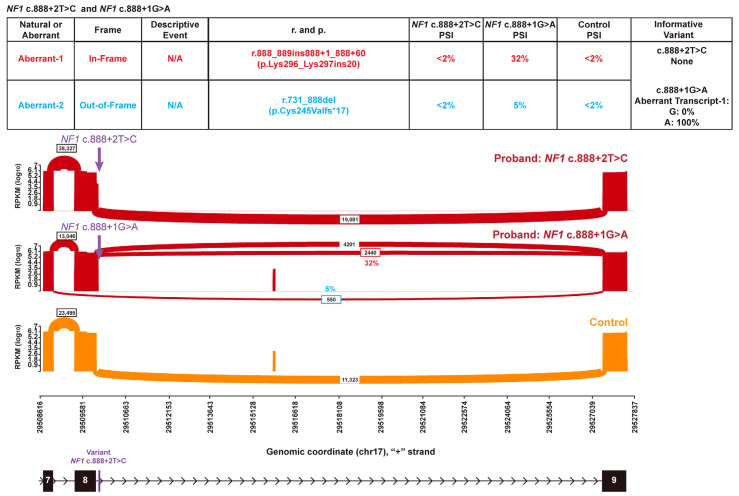
A canonical *NF1* variant exhibits no aberrant splicing. Sashimi plots illustrate the splicing of *NF1* Intron 8 and adjacent exons in a proband heterozygous for *NF1* c.888+2T>C (top red plot), a proband heterozygous for *NF1* c.888+1G>A (middle red plot), and a control individual (bottom orange plot). The number of RNA reads supporting a given splice event is indicated, and PSI is provided for the events representing aberrant splicing. Splice events with PSI < 2% have been filtered for clarity. The chromosomal position (hg19) is enumerated on the *x*-axis. The schematic below the plots depicts the genomic structure in the displayed region. The table above the Sashimi plots summarizes the detected splice events and aberrant splice events are color-coded to easily identify the event in the Sashimi plot and the table detailing the nature of the splice event.

*NF1* (NM_000267.3) c.888+2T>C (GRCh37 chr17:29509685-T-C) was identified in a patient who has a personal history of early-onset breast cancer and a family history of breast, bone and prostate cancers in first- and second-degree relatives. There were no reported features consistent with neurofibromatosis (OMIM #162200), a highly penetrant tumor predisposition syndrome caused by pathogenic variants in *NF1* [17]. Because the c.888+2T>C variant disrupts one of the two canonical nucleotide positions in the exon 8 donor splice site, this variant would typically be expected to result in a complete splice defect. However, RNA analysis did not detect any abnormal *NF1* transcripts above 2% PSI in the patient and WT controls (Figure 1), suggesting that this variant results in primarily normal transcript. In contrast, the *NF1* c.888+1G>A variant at the same canonical donor site results in substantial aberrant splicing (>30% PSI) from two events (*NF1* r.888_889ins888+1_888+60, p.Lys296_Lys297ins20 and *NF1* r.731_888del, p.Cys245Valfs*17). *NF1* c.888+1G>A is a well-established pathogenic variant that has been reported in numerous patients with neurofibromatosis in the literature, consistent with the RNA findings indicating a deleterious splice defect [18,19,20,21]. An individual heterozygous for *NF1* c.888+1G>A (GRCh37 chr17:29509684-G-A) produces an aberrant transcript containing 60 nucleotides of intron 8. This aberrant transcript exclusively harbors the aberrant A-allele (Figure 1). SpliceAI predictions for the two variants are consistent with the observed RNA impacts, with a donor loss score of 0.18 for the +2T>C variant contrasting with the 0.95 donor loss score for the +1G>A variant. Collectively, the RNA data from both variants supported a non-deleterious impact on splicing from *NF1* c.888+2T>C and precludes application of the very strong a priori line of pathogenic evidence (PVS1) typically applied to variants impacting either of the first two nucleotides within an intron.

### 3.2. Pseudo-Intron Creation

Although pseudo-exonization, in which a deep intronic sequence change simultaneously activates cryptic donor and acceptor sites to create a novel exon within the intron, is well described in the literature, the converse process, in which a sequence change leads to the concurrent activation of cryptic donor and acceptor sites within an exon, is not well documented. Here, we present an unusual example where a single nucleotide change creates a cryptic intron, through simultaneous activation of cryptic donor and acceptor sites within an exon.

**Figure 2:**
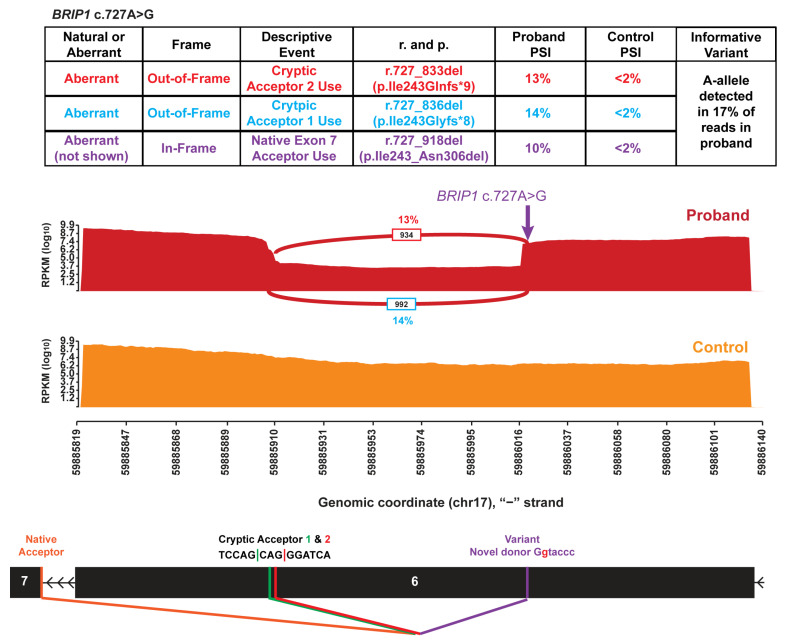
An exonic variant in *BRIP1* creates a pseudo-intron. Sashimi plots illustrate the splicing of *BRIP1* exon 6 in an individual heterozygous for *BRIP1* c.727A>G (top red plot) and a control individual (bottom orange plot). The number of RNA reads supporting a given splice event is indicated, and PSI is provided for the events representing aberrant splicing. Splice events with PSI < 2% have been filtered for clarity. The chromosomal position (hg19) is enumerated on the *x*-axis. The schematic below the plots depicts the genomic structure in the displayed region, with the locations of the multiple acceptor sites and novel donor site created by *BRIP1* c.727A>G indicated. The table above the Sashimi plots summarizes the detected splice events and aberrant splice events are color-coded to easily identify the event in the Sashimi plot and the table detailing the nature of the splice event.

*BRIP1* (NM_032043.2) c.727A>G (GRCh37 chr17:59886019-T-C) was identified in a female with a personal history of ovarian cancer and a family history of ovarian, breast, and stomach cancer in first- and third-degree relatives. The family also has a *BRCA1* nonsense variant that is not carried by the proband but that was identified in an affected first-degree relative. In the absence of a splice defect, the *BRIP1* c.727A>G variant is expected to generate a missense change, p.Ile243Val. In silico splice site analysis indicated that this variant creates a strong novel splice donor site (SpliceAI donor gain of 0.93) one nucleotide upstream of the variant. Use of this novel donor site is expected to result in the exclusion of this exon, including the missense change. However, in addition to the creation of a novel splice donor site, there was a concurrent prediction of activation of a cryptic splice acceptor site in exon six, 107 nucleotides downstream of the variant (SpliceAI acceptor gain of 0.49). RNA analysis identified three different partial exon skipping events that were not present in the controls (Figure 2). The cumulative PSI was 37% and the variant allele was only detected in ~10% (686/7071) of total RNA reads (Figure 2). Each event arises from the use of the novel cryptic donor site in coding exon 6, which pairs with three different existing acceptor sites: the native acceptor site for coding exon 7 (leading to *BRIP1* r.727_918del p.Ile243_Asn306del at 10.4% PSI), the aforementioned novel, cryptic acceptor site within coding exon 6 (leading to *BRIP1* r.727_836del p.Ile243Glyfs*8 at 13.6%); and another cryptic acceptor site just 3 nucleotides upstream of the aforementioned cryptic acceptor that was not predicted by SpliceAI (leading to *BRIP1* r.727_833del p.Ile243Glnfs*9 at 12.9% PSI). Use of the novel cryptic donor site in combination with either of the cryptic acceptor sites generated two smaller exons of 99 and 82–85 nucleotides, separated by a 106–109 nucleotide pseudo-intron, depending on which cryptic acceptor site was used. Collectively, these three abnormal transcripts are expected to disrupt BRIP1 protein function through either NMD or removal of a critical region of the protein.

### 3.3. Spliceosome Impact

More than 99% of introns in humans are spliced out of the pre-mRNA by the major U2 spliceosome; the minor U12 spliceosome is responsible for splicing only approximately 0.36% of all introns [22]. These spliceosomes have distinct snRNA compositions and recognize different consensus sequences for the previously described intron recognition motifs [23]. Here, we detail a variant that alters the spliceosome usage for a particular intron.

**Figure 3:**
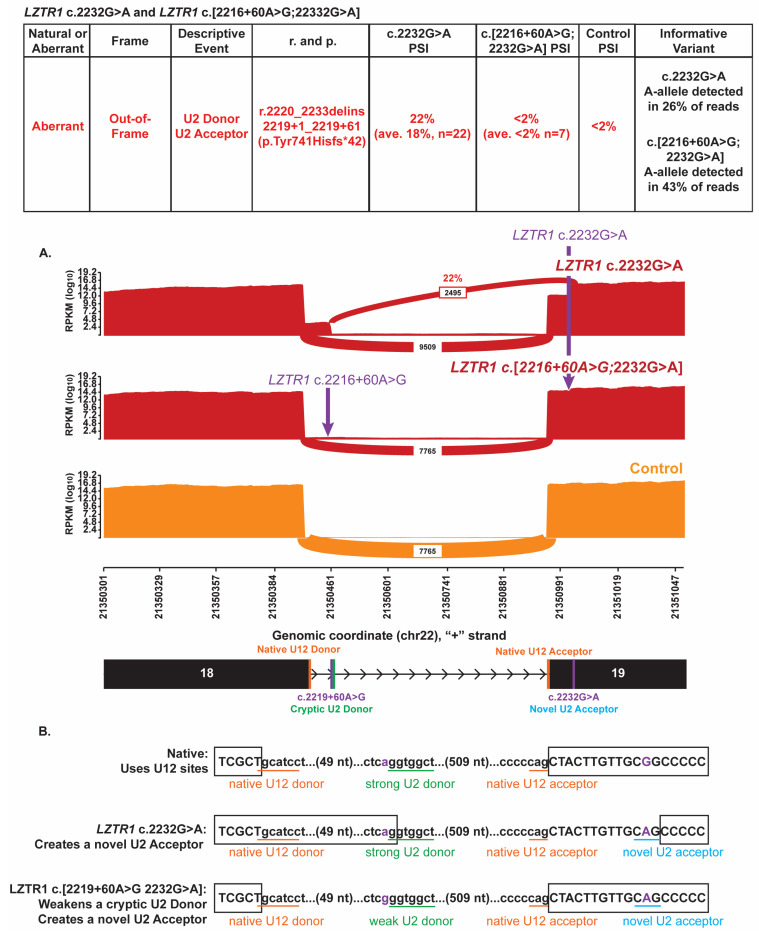
A variant in *LZTR1* influences spliceosome usage. (**A**) Sashimi plots illustrate the splicing of *LZTR1* intron 18 in a proband heterozygous for c.2232G>A p.Ala744Ala (top red plot), a proband heterozygous for c.2232G>A and homozygous for the common polymorphism c.2219+60A>G (middle red plot), and a control individual (bottom orange plot). The number of RNA reads supporting a given splice event is indicated, and PSI is provided for the events representing aberrant splicing. Splice events <2% PSI have been filtered for clarity. The chromosomal position (hg19) is enumerated on the *x*-axis. The schematic below the plots depicts the genomic structure in the displayed region, with the locations of the benign polymorphism, the cryptic U2 donor, and the novel U2 acceptor indicated. The table above the Sashimi plots summarizes the detected splice events and aberrant splice events are color-coded to easily identify the event in the Sashimi plot and the table detailing the nature of the splice event. (**B**) Schematic detailing the utilized splice sites for a c.2232G>A allele, a c.2219+60A>G; c.2232G>A allele, and a control allele. Capital letters indicate reference exonic sequence, lower case letters delineate reference intronic sequence, and black boxes depict exon ends in each scenario (for splice events >2% PSI). Native U12 splice sites are underlined in orange, the cryptic U2 donor sites in green, and the novel U2 acceptor site in blue. The c.2232G>A variant and c.2219+60A>G polymorphism are denoted by purple text.

*LZTR1* (NM_006767.3) c.2232G>A (p.Ala744Ala; GRCh37 chr22:21350997-G-A) is a synonymous variant in exon 19. *LZTR1* has complex heritability as primarily an autosomal recessive, loss-of-function disease causing Noonan syndrome but also with rare domain-restricted, dominant-negative variants causing Noonan syndrome in an autosomal dominant fashion. Additionally, pathogenic alterations in *LZTR1* combined with specific somatic alterations cause Schwannomatosis. This variant has been detected over 160 times in this clinical cohort and has neither been identified with other germline pathogenic *LZTR1* variants nor in an individual reporting features of Noonan syndrome or Schwannomatosis. This variant creates a strong novel U2 acceptor site (SpliceAI acceptor gain of 0.99). As intron 18 in *LZTR1* is normally spliced via the minor U12 spliceosome, it was surprising to find that *LZTR1* c.2232G>A is associated with a significant amount of aberrant splicing (Figure 3A). However, this aberrant splicing is observed only in a subset of heterozygotes (76% (22/29 heterozygotes)). In that subset of individuals, the novel acceptor stie is utilized in conjunction with a quiescent, upstream cryptic U2 donor site located within intron 18 at *LZTR1* c.2219+61 (Figure 3B; GRCh37 chr22:21350462). The average PSI for these heterozygotes is 18% +/− 5%, with the variant nucleotide present in an average of 26% of total RNA reads (based on analysis of 11 probands’ RNA data). The resulting transcript contains a frameshift and is expected to undergo NMD (*LZTR1* r.2220_2233delins2219+1_2219+61 p.Tyr741Hisfs*42). In the remaining 24% of heterozygotes (7/29), very little aberrant splicing was detected (average PSI 2% +/− 1.6%), with the variant nucleotide present in an average of 43% of total RNA reads (based on analysis of 4 probands’ RNA data). Inspection of the DNA sequence around the cryptic donor at c.2219+61 in all heterozygotes revealed that individuals in the low-PSI group were homozygous for the common polymorphism *LZTR1* c.2219+60A>G (GRCh37 chr22:21350461-A-G; gnomAD v2 allele frequency 0.781 [https://gnomad.broadinstitute.org/variant/22-21350461-A-G?dataset=gnomad_r2_1, GnomAD v2.1.1., accessed on 10 November 2025], while all individuals in the high-PSI group were either heterozygous (*n* = 17) or wildtype (*n* = 5) for that polymorphism. The c.2219+60A>G polymorphism weakens the cryptic U2 donor site at c.2219+61 (predicted SpliceAI donor loss of 0.66), explaining why the cryptic site is not utilized in the low-PSI group homozygous for the polymorphism. As such, the U2 spliceosome is typically not utilized for intron 18 splicing in *LZTR1* c.2232G>A heterozygotes where the polymorphism occurs in cis with the variant and normal U12 spliceosome-dependent splicing predominates (Figure 3B). In c.2232G>A heterozygotes lacking the in cis polymorphism, the U2 spliceosome competes with the U12 spliceosome, resulting in the observed mix of normal and aberrant splicing derived from the variant allele.

### 3.4. NMD-Escaping

Nonsense and frameshift variants are usually expected to undergo NMD. Variants in loss-of-function genes are therefore ascribed the a priori very strong line of pathogenic evidence, PVS1. Here, we describe two PVS1-eligible variants that escaped NMD for unexpected reasons.

**Figure 4:**
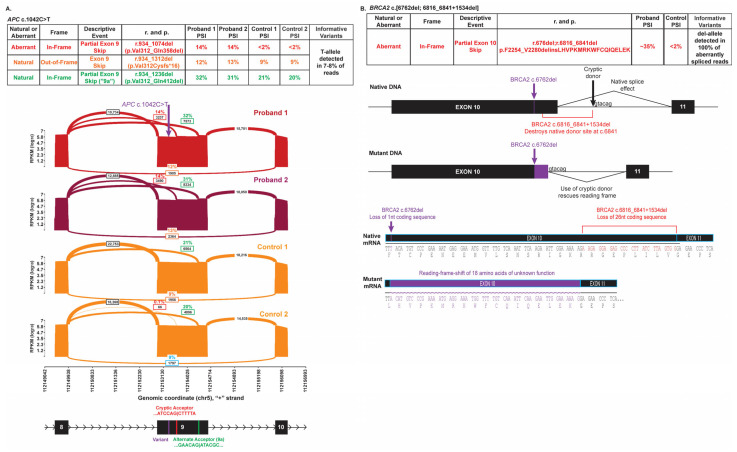
Splicing impacts rescue transcripts that would otherwise undergo to NMD. (**A**) Sashimi plots illustrating splicing of exons 8 through 10 in probands 1 and 2 heterozygous for the *APC* c.1042C>T, p.Arg348Ter (top red and burgundy plots) relative to controls 1 and 2 (bottom orange plots). The number of RNA reads supporting a given splice event is indicated, and PSI is provided for the events representing aberrant splicing. Splice events <2% PSI have been filtered for clarity. The chromosomal position (hg19) is enumerated on the *x*-axis. The schematic below the plots depicts the genomic structure in the displayed region, with the locations of the variant, the strengthened cryptic donor site, and the alternate donor site indicated. The table above the Sashimi plots summarizes the detected splice events and aberrant splice events are color-coded to easily identify the event in the Sashimi plot and the table detailing the nature of the splice event. (**B**) A schematic diagram depicts the splicing of *BRCA2* exons 10 and 11 in native DNA and DNA with *BRCA2* c.6762del; c.6816_6841+1534del (top) along with the resulting RNA transcript sequence (bottom). Exons are represented as boxes and introns depicted as lines. The locations of the two in cis variants as well as the cryptic donor that is utilized are indicated with respect to the native genomic structure and RNA sequence, and the position of the cryptic donor is shown as well. The table above the schematic summarizes the detected splice events.

*APC* (NM_000038.5) c.1042C>T (p.Arg348Ter; GRCh37 chr5:112154771-C-T) has been identified in six probands over the age of 50 who did not report a clinical history of polyposis or other *APC*-associated phenotypes (OMIM #175100). On follow up, two individuals were confirmed to be on a 10-year colonoscopy schedule suggesting that there was not a significant polyp burden in either of them. Personal histories included uterine cancer, breast cancer, and prostate cancer in three individuals while the other three had no personal history of cancer. The collective family histories in first-degree relatives included breast, cervical cancers. This variant lies within the 5′ end of *APC* exon 9 in the region of the exon that is alternatively spliced in a naturally occurring, in-frame transcript called 9a [24]. Pathogenic variants within the 5′ alternatively spliced region of *APC* exon 9 are associated with attenuated familial adenomatous polyposis (FAP), which generally effects a substantial polyp burden but is considered reduced relative to classic FAP. This suggests a dosage effect where the greater the loss of wild-type transcript, the greater the phenotypic impact/polyp burden [25]. A SpliceAI acceptor loss score of 0.26 and an acceptor gain score of 0.24 are associated with this variant. RNA analysis demonstrated that the *APC* c.1042C>T alteration, which creates a PTC, is associated with an increase in the amount of the naturally occurring, in-frame alternate transcript 9a (*APC* r.934_1236del p.Val312_Gln412del), expressed at approximately 31% and 32% PSI in two heterozygotes versus 20% and 21% PSI in controls (Figure 4A), though the utilized acceptor site was not predicted by spliceAI. However, RNA analysis also confirmed the use of the predicted alternate splice acceptor site introduced by the variant at 14% PSI in both cases. The use of this site leads to a novel, in-frame transcript that removes part of exon 9 (r.934_1074del p.Val312_Gln358del) (Figure 4A). Both the novel transcript and the naturally occurring 9a transcript result in in-frame events that splice out the nonsense variant, creating a pool of variant-derived transcripts that are expected to escape NMD. Ultimately, the nonsense alteration is only present in approximately 7–8% (1894/26231; 1839/22549) of total RNA reads, which may not be enough to cause disease. As such, the PVS1 code for an a priori loss-of-function variant was not ascribed to this alteration as alternative splicing may result in a functional protein.

*BRCA2* (NM_000059.3) c.6762del (GRCh37 chr13:32915253-TT-T) and c.6816_6841+1534del (GRCh37 chr13:329165308-32916867) are two variants in *BRCA2* coding exon 10 that independently would be predicted to undergo NMD. The former single nucleotide deletion causes a frameshift and premature termination; the latter, larger deletion encompasses the entire canonical splice donor site of coding exon 10 and unexpectedly also creates a strong donor site at the novel junction (SpliceAI donor gain of 0.53). The resulting splicing impact of the large deletion is also a frameshift (r.6816_6841del) resulting in a PTC. However, these two variants were confirmed in cis in two apparently unrelated female probands, one in her 50s and one in her 30s. Each individual submitted a personal or relative’s report from another clinical laboratory reporting two pathogenic variants in *BRCA2*. Neither individual had a personal history of cancer or a family history of cancer in first-degree relatives. Due to the presence of both the single nucleotide deletion and the frameshifting splice impact on the same allele, the total effect (*BRCA2* r.6762del; r.6816_6841del p.Phe2254_Val2280delins18), which is present in ~35% of RNA reads, preserves the reading frame of this transcript and results in a predicted in-frame loss of 26 amino acids and an insertion of 18 amino acids (Figure 4B). Due to the restored reading frame and the unknown functional impact of the new amino acid sequence in the affected region, the PVS1 code for an a priori loss-of-function variant was not ascribed to this variant as it may result in rescue of function.

### 3.5. Cryptic Pyrimidine Tract Modifications

One key component of a splice acceptor site is the polypyrimidine tract, which promotes the assembly of the spliceosome for intron excision. Due to its variable length and composition, it is difficult to assess how any particular nucleotide change in the polypyrimidine tract may affect splicing in the absence of functional RNA data. Here, we report alterations which appear to result in the strengthening of a polypyrimidine tract that, in turn, activates a cryptic acceptor leading to aberrant splicing.

**Figure 5:**
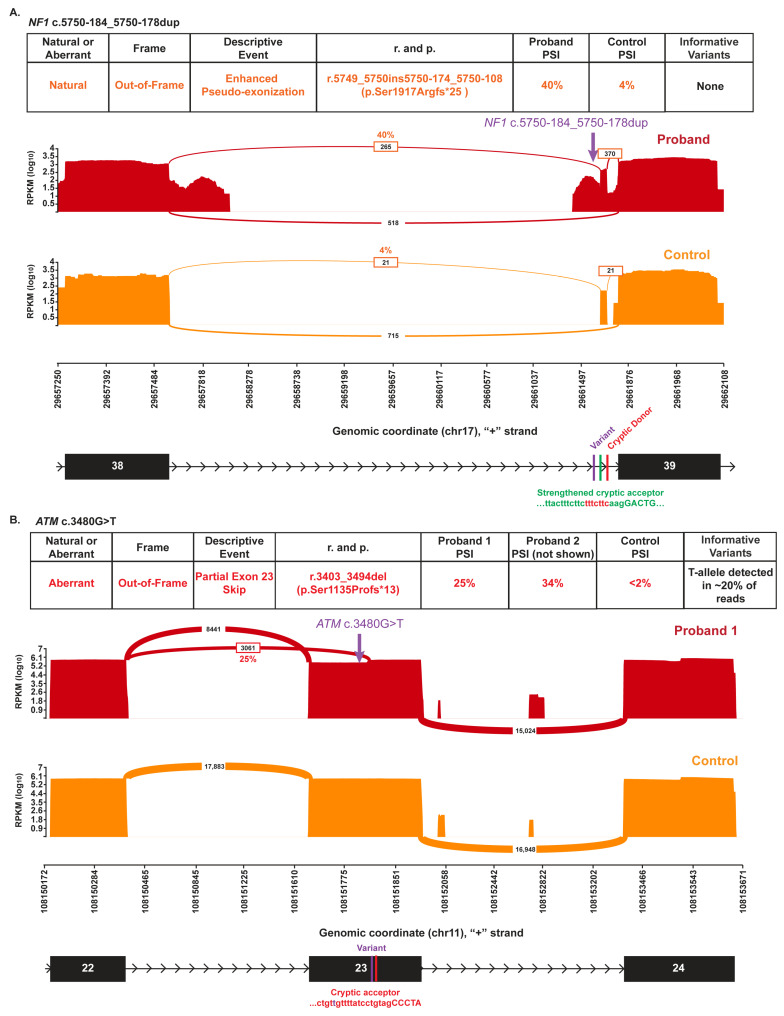
Variants impacting the pyrimidine tract of cryptic splice acceptors cause aberrant splicing. (**A**) Sashimi plots illustrate the splicing of *NF1* intron 38 in a proband heterozygous for *NF1* c.5750-184_5750-178dupTTTCTTC (top red plot) and a control individual (bottom orange plot). (**B**) Sashimi plots illustrate the splicing of exons 22 through 24 in a proband heterozygous for *ATM* c.3480G>T (p.Val1160Val) (top red plot) and a control individual (bottom orange plot). In all Sashimi plots, the number of RNA reads supporting a given splice event is indicated, and PSI is provided for the events representing aberrant splicing. Splice events with PSI < 2% have been filtered for clarity. The chromosomal position (hg19) is enumerated on the *x*-axis. The schematics below the plots depict the genomic structure in the displayed region, with the locations of the variant and the relevant cryptic splice sites indicated. The tables above the Sashimi plots summarize the detected splice events and aberrant splice events are color-coded to easily identify the event in the Sashimi plot and the table detailing the nature of the splice event.

*NF1* c.5750-184_5750-178dup (GRCh37 chr17:29661671-C-CTTTCTTC) was identified in a child with multiple café-au-lait spots, suspected subcutaneous neurofibromas, axillary freckles and macrocephaly. RNA analysis demonstrated that this alteration is associated with increased expression of *NF1* r.5749_5750ins5750-174_5750-108 p.Ser1917Argfs*25 relative to a wild-type control (Figure 5A). In the wild-type control, the r.5749_5750ins5750-174_5750-108 transcript, present at 4% PSI, represents low-level, naturally occurring, alternative splicing arising from the use of cryptic donor and acceptor sites within intron 38. In the proband, however, the transcript is expressed at 40% PSI. The substantially higher expression is likely attributable to the increased pyrimidine content introduced by the duplication of TTTCTTC within a polypyrimidine tract proceeding the cryptic acceptor at *NF1* c.5750-174 (GRCh37 chr17:29661682), which results in the strengthening of the cryptic acceptor site (SpliceAI acceptor gain of 0.59). The strengthening of the cryptic acceptor site leads to increased production of this naturally occurring NMD-prone transcript and is expected to result in a loss of protein expression and development of neurofibromatosis phenotype in the proband. Although the utilized cryptic donor site is also predicted with a SpliceAI donor gain of 0.42, this likely reflects the activation of the donor site due to the strengthened acceptor site rather than a direct impact of the variant on the cryptic donor site itself.

*ATM* (NM_000051.3) c.3480G>T (p.Val1160Val; GRCh37 chr11:108151799-G-T) is a synonymous alteration detected in four apparently unrelated females age 39–71. One individual had ovarian cancer, two had uterine cancer, and one had unspecified stomach cancer. Collective family history included prostate cancer, breast cancer, and colorectal cancer in first degree relatives. In the absence of an RNA splicing impact, synonymous variants are not expected to cause disease as they do not change the amino acid sequence of the protein. RNA analysis for this alteration identified the presence of an NMD-prone transcript, *ATM* r.3403_3494del p.Ser1135Phefs*13, at 25% and 34% PSI in the two probands. This transcript was absent in the control (Figure 5B). This variant, a purine (G) to pyrimidine (T) transversion at c.3480, increases the pyrimidine content of a polypyrimidine tract upstream of a cryptic acceptor at c.3494 (GRCh37 chr11:108151813), thereby strengthening and activating the utilized cryptic acceptor site (SpliceAI acceptor gain of 0.2). The variant did not, however, have a complete impact on splicing, as normally spliced RNA reads derived from the variant allele constituted 19% (2103/11387) and 21% (328/1561) of total RNA reads in the two probands.

## 4. Discussion

Understanding the nature and measuring the magnitude of splice effects is critical to variant interpretation and clinical reporting of genetic test results. Despite great progress that allows reasonably confident use of in silico splice tools, there remain nuanced examples of unexpected splicing that underscore the sensitive and complex nature of the splicing process. This makes careful consideration of splice predictors (including their limitations) and adoption of universal RNA testing paramount to accuracy. This work describes seven examples of unexpected RNA findings that further expand our understanding of clinically important RNA splice considerations.

The concept that +2T>C changes may not have a complete splice effect is not novel [14,15]; however, it is also not well disseminated in the variant interpretation community, and the breadth and ability to predict which +2T>C changes break from the convention of a near-complete splice defect has yet to be comprehensively ascertained. The *NF1* c.888+2T>C variant is a poignant example as neurofibromatosis type-1 is a high penetrance disease caused only by pathogenic variants in *NF1*. The high quality, patient-derived splice assay supporting a lack of substantive splice defect, coupled with a lack of clinical features, forms a credible interpretation for this variant as benign. Of note, the SpliceAI score for this variant predicted a reduced probability of a splice effect (0.18) relative to other canonical splice site alterations which trend between 0.7 and 1.0. This example is consistent with a recent study that demonstrated that SpliceAI is able to detect anomalous +2T>C changes bioinformatically with a higher success rate than most other tools [26].

While sequence changes that activate cryptic exons have been reported as disease causing, sequence changes that lead to cryptic intron activation appear to be rarer [27]. Cryptic intronization may be influenced by the length of the cryptic intron as well as the presence of normally dormant splice motifs, including the polypyrimidine tract and branch point. Human intron length varies but has median lengths of around 1000 nucleotides and a reported minimum intron length of ~100 nt [28]. Given that the average human exon is <200 nucleotides [29], intronization can be expected to be a rare event and only result in small, novel introns [30]. In support of this, the cryptic introns activated by *BRIP1* c.727A>G are 106–109 nucleotides long, which is small but consistent with the cited minimum intron size. Notably, there is a putative polypyrimidine tract flanking the cryptic acceptor sites activated by the *BRIP1* variant that is likely contributing to the recognition of these acceptor sites [30].

Most introns are spliced exclusively by a single spliceosome (U2 or U12), but on rare occasions an intron will contain two complete sets of splice sites—one set each for the U2 and U12 spliceosomes—which is referred to as a U2/U12-type twintron [31,32,33]. In the course of these RNA studies, a variant that completes a twintron by creating a novel U2 acceptor site was discovered. These U2 sites are only used in the context of the reference allele for a common single nucleotide variant at *LZTR1* c.2219+60A>G, further complicating the prediction and interpretation of the variant impact. To our knowledge, this is the first report of a human variant resulting in a spliceosome switch. Given the incomplete nature of the aberrant splice event resulting from the spliceosome switch, the clinical relevance of this spliceosome switch is still unclear.

The concept of alternative splicing providing a putative rescue mechanism for loss-of-function variants is increasingly considered in variant interpretation. One example is in *BRCA1* where a commonly observed isoform lacks two constitutive exons leading to an in-frame, NMD-escaping event that is clinically sufficient [34]. Another example of the rescue of nonsense variants by alternative splicing is described in the thyroglobulin gene for exon 22 [35]. These examples are similar to the *APC* c.1042C>T (p.Arg348Ter) alteration where the in-frame loss of portions of exon 9 may mitigate the clinical impact. The *BRCA2* c.6762del; c.6816_6441+1534del variant provides another example. In this case, a functional splice donor site unexpectedly formed at the junction of the large deletion, and use of that donor site in conjunction with the upstream frameshift restored the reading frame. Another mechanism whereby the presence of a nonsense variant evokes alternative splicing events that rescue the nonsense effect is nonsense-associated alternative splicing (NAS) [36]. It remains to be tested as to whether NAS is an explanation for the apparent up-regulation of the naturally occurring alternative transcript 9a, which could further contribute to nonsense rescue in the *APC* example. These examples highlight the importance of evaluating splicing predictions and even better actual RNA data, for all variant types including haplotypes, not just those with a high spliceogenic potential.

Lastly is the example of small modifications to the polypyrimidine tract. The polypyrimidine tract may exist simply to deplete the intronic sequence of incidental AG dinucleotides that may otherwise serve as alternative (and deleterious) canonical residues within a consensus splice acceptor site [37,38]. The length of the polypyrimidine tract may be important; however, the exact composition of the tract is more flexible. In vitro studies have shown that the number of consecutive uridine residues combined with the distance from the canonical AG site are important factors for optimal splicing such that small uridine stretches need to be immediately adjacent to the AG acceptor site in order to be competitive [39]. This may be the case for the *NF1* c.5750-184_5750-178dup, which duplicates a T/C rich sequence (TTTCTTC) causing aberrant splicing at an AG site just two nucleotides downstream. The *ATM* c.3480G>T alteration changes a purine to a pyrimidine near a stretch of pyrimidines where a polypyrimidine tract would be expected based on its location relative to the cryptic acceptor site. In the context of the cryptic acceptor site, which matches the consensus (C/T)AG acceptor site motif, it appears that the resulting sequence out-competes the native splice acceptor site. The native splice acceptor site contains a polypyrimidine tract with a uracil at 18 of 21 nucleotides with only one nucleotide separating the AG from the tract, which should constitute a strong splice pyrimidine tract. However, the nucleotide at the -3 position of the native acceptor site is an adenine, which deviates from the consensus (C/T)AG acceptor site sequence and may explain why the cryptic acceptor site with a strengthened pyrimidine tract is preferred. Nevertheless, it is important to note that it is possible that the c.3480G>T variant is mediating its splicing effect via an impact to an unrecognized splice enhancer or silencer rather than via pyrimidine tract strengthening.

Of note, when in silico splice tool predictions are considered in the context of the observed RNA impact, the scores can be retrospectively aligned with the observed impact for many of these variants. However, combining the various donor and acceptor loss and gain scores in such a way to generate the correct r. prediction in the absence of RNA data is error-prone, particularly for the unusual types of RNA findings described herein. Predicting the degree to which splicing will be impacted from the SpliceAI scores alone is also unreliable. Thus, in silico splice predictions on their own are insufficient to reliably predict splicing outcomes, underscoring the necessity of RNA-based validation.

For patient-derived, blood-based RNA methodologies it’s possible that some aberrant transcripts will go undetected due to technical limitations or disparate nonsense-mediated decay activity among different transcripts and/or in different patients. In addition, the splicing profiles presented herein may not be representative for several reasons: acquired mutations-particularly in the case of clonal hematopoiesis of indeterminant potential (CHIP)-can disproportionally affect some genes (e.g., *NF1*) and mosaicism in the blood may lead to underrepresentation in inability to detect resulting low-level transcripts. In addition, splicing profiles in the blood may not be representative of the splice profile in the tissue where phenotype manifests. Caveats and limitations apply to all diagnostic testing and those listed herein are expected to impact very few tests. Nonetheless, it’s important to couch these limitations by amassing multiple types of variant interpretation evidence, which is a central tenet of any high-quality variant interpretation scheme. Although it is outside the scope of this work which aims to present variants with unexpected RNA findings, the interpretation of these variants should include not only RNA observations but also other evidence types such as, population frequency, patient/family phenotype (which is often of limited utility for cancer genes as provided herein), functional studies, and bioinformatics.

## 5. Conclusions

Overall, this collection of spliceogenic examples underscores the importance of three foundational concepts related to spliceogenic variants. First, some splice effects are unexpected and difficult to predict and interpret in the context of a simplistic view of splicing. Second, evaluation of RNA is the best practice to interpret variants identified in patients. Finally, the provision of an a priori very strong line of evidence (usually ACMG/AMP PVS1 code) to some variants based on the variant type can be misguided in the absence of the consideration of splice anomalies. These results expand knowledge of clinically important splicing mechanisms and highlight the complexity of the RNA splicing process. This work shows that variants can have unusual and unanticipated impacts on splicing such that predictions based on splicing algorithms, the type of variant, and a general understanding of splicing may differ from the observed RNA transcripts. For the variants presented here, paired DNA and RNA testing uncovered splice events that altered variant interpretation, underscoring the utility of RNA testing in a clinical diagnostic setting.

## Data Availability

All data, generated or analyzed during this study, including ethics-compliant deidentified and/or aggregate clinical information, are included in this published article.

## Abbreviations

ACMG/AMP	American College of Medical Genetics/American College of Medical Genetics
CHIP	Clonal Hematopoiesis of Indeterminant Potential
FAP	Familial Adenomatous Polyposis
NAS	Nonsense-Associated Alternative Splicing
NMD	Nonsense-Mediated Decay
PSI	Percent Splicing Index
PTC	Premature Termination Codon
PVS1	Pathogenic Very_Strong 1
RT-PCR	reverse transcriptase polymerase chain reaction sequencing
VUS	Variant of uncertain significance
WT	Wildtype
